# The food matrix as a confounder in diet‒microbiome studies: methodological challenges and research gaps

**DOI:** 10.1080/29933935.2026.2671724

**Published:** 2026-05-13

**Authors:** Erin O'Sullivan, Olivia Solano, Julia S. Oliveira, Abigail J. Johnson, Sushil Dhital, Prasanth K. S. Pillai, Fernanda Dias, Job Ubbink, Annie W. Lin, Levi Teigen

**Affiliations:** a Department of Food Science and Nutrition, University of Minnesota, St. Paul, MN, USA; b The Hormel Research Institute, University of Minnesota, Austin, MN, USA; c Division of Epidemiology and Community Health, School of Public Health, University of Minnesota, Minneapolis, MN, USA; d Riddet Institute, Massey University, Palmerston North, New Zealand; e Department of Chemical and Biological Engineering, Monash University, Clayton, VIC, Australia

**Keywords:** Food matrix, gut microbiome, food processing, food structure, diet–microbiome

## Abstract

Interactions of structural and physicochemical properties of food (e.g. texture, viscosity, and solubility), known as the food matrix, are primary drivers of host digestive kinetics. While the impact of matrix-driven variability on nutrient bioaccessibility, glycemic response, and caloric absorption is well documented, these dynamics are often overlooked in diet-microbiome research. Adequately modeling the spatiotemporal availability of microbial substrate is essential to understanding how dietary patterns impact the microbiome throughout the gastrointestinal tract. This narrative review 1) synthesizes current evidence on how food processing and structure affect human digestion and absorption of macronutrients; 2) illustrates how host digestive kinetics impact forms and quantity of substrate delivered and available to the gut microbiota; and 3) identifies challenges and knowledge gaps in current diet–microbiome research regarding food structure. The challenges and knowledge gaps discussed here call for in vivo models that can better model microbial substrate availability throughout the gastrointestinal tract to improve our understanding of diet–microbiome interactions.

## Introduction

The physiochemical architecture of food (i.e. the food matrix) plays a critical role in host digestion and absorption kinetics. It is well established that the food matrix independently influences human digestive kinetics and physiological endpoints, such as glycemic index and fecal energy losses.^1-3^ Human diet–microbiome research has largely been unable to account for food matrix-related effects, which varies due to the degree of processing, plant species, tissue, harvesting time, and other environmental factors.^4^ There are several methodological challenges in capturing the complex and dynamic interactions of human dietary patterns, food matrix, and digestive physiology.^5-7^

Common approaches, specifically nutrient-level analyzes, provide important information about diet–microbiome relationships. One frequent approach includes investigating the central role of dietary fiber in supporting gut microbiome health.^8-10^ However, diets that have similar nutrient compositions are often divergent in the types of foods and their physical structure.^11-14^ Illustrating this point is the recent seminal work by Tanes et al. characterizing recovery of the human gut microbiome following antibiotic exposure at high, moderate, and fiber-free fiber levels. The fiber-free diet was exclusively liquid, making it difficult to know what microbiome changes are fiber-specific and which are related to altered substrate kinetics with a liquid diet.^9^

Transitioning toward a matrix-centered perspective requires methods that capture and model features of the food matrix that impact absorption and fermentation kinetics along the gastrointestinal tract.^13^^,^^15-17^ These factors often include consideration of particle size, cell composition, viscosity, and solubilization.^18^ Understanding the impact of matrix-related alterations on nutrient bioaccessibility for both host and gut microbiota is pivotal to advance diet-microbiome research.

This narrative review aims to discuss the influence of the food matrix on digestive kinetics and to highlight potential implications for microbial substrate availability. We first review how food processing and preparation methods alter the food matrix and the macroscopic structure of macronutrients. Then, we discuss the implications of an altered food matrix on microbial substrate availability throughout the length of the gastrointestinal tract. Finally, we present the knowledge gaps and challenges to establish future directions for incorporating food matrix as a variable in diet-microbiome research.

## Methods

A nonstructured, exploratory search strategy was conducted in PubMed and Web of Science to identify studies evaluating the food matrix, digestion kinetics, and the gut microbiota. Human models were prioritized, but studies using in vitro or animal models were also included. Narrative reviews were consulted as support material for well-established ideas related to food processing. The quality of this narrative review was assessed using the Scale for the Assessment of Narrative Review Articles (SANRA) (Supplementary Material).

### Food processing as a determinant of food matrix structure

Food processing operations are often used to modify complex food matrices that encapsulate nutrients in plant and animal tissues. Common food processes include milling, grinding, homogenization, emulsification, cooking, and fermentation, all of which can affect nutrient release, accessibility, and digestion. Disrupting or modifying existing structures and physiochemical properties of food alters enzyme access and nutrient bioavailability.^18^ The following subsections examine the effect of food processing on the bioavailability of macronutrients, as they represent a primary source of nutrients for both the host and the microbiome. Given the focus on macronutrients, this section will not address other components in food that are present in smaller quantities (e.g. vitamins, minerals, phytonutrients, and additives). Nevertheless, it is recognized that these components may also influence the digestion of macronutrients and/or have distinct microbiome effects.

### Dietary carbohydrates

**Figure 1.**
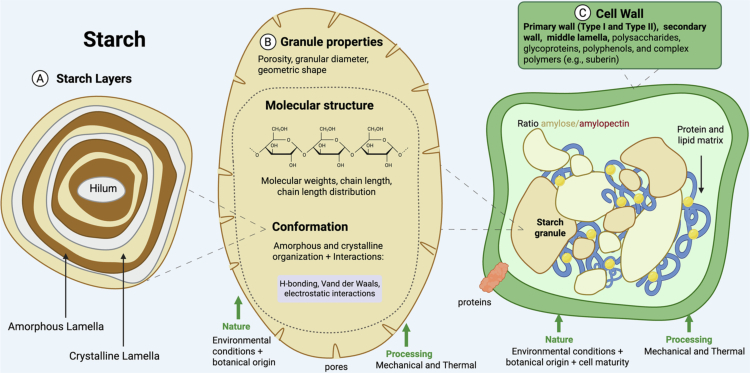
Characteristics of the plant cell wall and starch granules that determine physiochemical and functional properties of plant-based foods. Starch properties including gelatinization, swelling, retrogradation, and pasting are a result of molecular structure and conformation of starch (A), which are vulnerable to different processing techniques and variations in nature.^22^ The availability of starch for digestion by α-amylase depends on starch granule properties (B) and the integrity of the cell wall (C), which contains indigestible fiber. Within the cell wall, starch is stored as granules, in which starch molecules (amylose and amylopectin) form alternating layers of amorphous and crystalline arrangements as observed in “Starch Layers” (A). Food processing techniques aim to manipulate starch properties by altering various levels of structure. For example, milling can damage the cell wall and granular structure, while starch gelatinization and retrogradation can disrupt the layered amorphous‒crystalline structures of the starch granule to affect digestibility. This diagram is intended as a general representation of starch-containing cells and is not representative of structural variability across different plant-based food sources.^22–26^

Carbohydrates encompass many different forms in the diet, including low molecular weight sugars, starch, and fiber. In plants, starch is stored intracellularly as starch granules, consisting of alternating amorphous and crystalline layers of amylose and amylopectin. Starch granules are surrounded by the cell wall in the endosperm of cereals, or cotyledon of legumes and tubers. The efficiency of carbohydrate digestion and absorption depends on enzymatic access to starch,^19^^,^^20^ and binding of enzymes to the starch granule surface.^21^ Structural components, however, can vary depending on the plant species,^22^ as well as food processing treatments, which disrupt the integrity of cell walls and modify the starch structure (Figure 1A–C).^23-26^

Molecular modification of starch granules impacts starch properties, including solubility, swelling, pasting (the rupture of granules leading to increased apparent viscosity), and ultimately, digestibility.^22-24^^,^^27^^,^^28^ Thermal processing, like steaming or boiling, can be used to increase palatability and digestibility of starch-containing foods by facilitating gelatinization and granule swelling. This alteration provides greater surface area for enzymes to digest starch, increasing its bioaccessability.^29^ In contrast, the partial recrystallization of starch molecules that occurs during cooling following heating, known as starch retrogradation, provides physical and steric hindrance within the glucan chains. This process results in type 3 resistant starch (RS3), which restricts enzyme access and impedes digestion.^30^^,^^31^ High-heat methods combined with a fat source, such as stir-frying, can lead to the formation of resistant starch and other enzyme-resistant complexes.^29^ This occurs through the formation of amylose–lipid complexes (type 5 resistant starch) and lipid coating effects, making the starch physically inaccessible to amylases (type 1 resistant starch).^29^

Fermentation and germination also alter the molecular structure of starch prior to thermal processing (e.g. baking). Both processes partially hydrolyze amylose and long branch chain amylopectin reducing molecular weight and disrupting crystalline and amorphous regions within the starch granule. In the case of fermentation, an acidic environment promotes granule swelling, surface erosion, and reorganization of starch polymers, which may increase the digestibility of fermented starch products.^32^^,^^33^

Thermal treatments (i.e. cooking) and mechanical processing (e.g. chewing, milling or mechanical grinding) induce structural modifications, mostly through disruption of the cell walls. An intact cell wall encapsulates starch granules, rendering them inaccessible to digestive processes if left in their native state. Disruptions to the cell wall remove the physical barriers and expose starch granules to the host and microbes,^34^^,^^35^ specially in the milling techniques that reduce cereal grains and legumes into flours.^36^

### Dietary fat

Dietary lipids encompass a diverse group of molecules, primarily triglycerides, followed by phospholipids, sterols, and other lipid-soluble compounds.^37^^,^^38^ Triglycerides predominantly serve as an energy reservoir in adipose tissue, while sterols (e.g. cholesterol) serve as structural membrane components or are used in the synthesis of steroid hormones. Lipids are classified by their state at room temperature: solid fats, typically from animal sources, and liquid oils, typically from plant sources.

While host-specific factors dictate the efficiency of upper gastrointestinal digestion, the food matrix serves as a determinant of the rate, form, and composition of lipid substrates that reach the distal gut. Industrial and domestic processing induces compositional, structural, and spatial changes within the food matrix, thus altering lipid bioavailability.^39^ This manipulation of the matrix primarily focuses on modifying fatty acid saturation levels and chain lengths to produce lipids with tailored molecular architectures and functional properties.^40^^,^^41^ Emulsification is a common approach that controls lipid organization, droplet size, and interfacial stability. An emulsion is formed by applying mechanical force and/or emulsifiers to combine two immiscible liquids. Foods such as sauces or milk are considered emulsions since tiny lipid droplets are stabilized by protein and starches, allowing lipid droplets to be delivered at a controlled rate and specific form in the body. Similar to carbohydrates, industrial techniques, like high-pressure homogenization and extrusion, can also be used to disrupt plant cell walls. This action can promote the transfer of lipophilic bioactive compounds into oil droplets, increasing their potential bioaccessibility.^42^

Lipid droplet size and interfacial composition are critical determinants of digestion kinetics and nutrient bioaccessibility.^38^^,^^43-45^ While smaller lipid droplets are generally more rapidly digested due to increased surface area,^44^ excessively fine particles have been shown to delay absorption.^45^ Further, processing-induced changes in lipid physical structure may influence oxidative stability, leading to the formation of lipid oxidation products (LOPs).^46^ Reduced droplet size and increased interfacial area can accelerate lipid oxidation by increasing exposure to pro-oxidant species and interfacial reactions, particularly in emulsified systems. The nature of the emulsifier, the presence of transition metals (e.g. iron, copper), and the distribution of antioxidants at the oil-water interface further modulate oxidative pathways.^47^ Ultimately, the interfacial design and droplet morphology of dietary fats influence the efficiency of micelle formation and lipid absorption in the small intestine.

### Dietary proteins

Protein, at the absorbable level, is a three-dimensional structure composed of an amino acid sequence.^48-50^ Within a food matrix, proteins can be found encapsulated within plant cell walls (e.g. legumes) or integrated into complex fibrous structures in animal muscle cells or casein-stabilized micelles in dairy. There are various types of proteins in food, each acting in accordance with their molecular and microscopic nature and the biochemical environment in which they reside in.^51^ Before food is ingested, various processes can alter protein structure, directly impacting their digestive kinetics.^52^

Industrialized applications commonly extract amino acids from different protein sources as ingredients to form new food protein complexes^53^; while traditional cooking, depending on the method (e.g. degree of heat), can modify amino acid side chains and exposure of peptide bonds.^54-57^ Thermal processing can also lead to the formation of dietary advanced glycation end products (dAGEs), known as melanoidins, through nonenzymatic reactions between protein and reducing sugars, lipids, or nitrogen bases.^58^^,^^59^ This process is known as the Maillard reaction, which contributes to stronger flavor and increased palatability and in protein-rich foods.^59^

Proteins within the same food matrix can exhibit vastly different digestive behaviors. For example, in vitro digestion of homogenized whole milk shows that caseins tend to coagulate in the acidic environment of the stomach, slowing gastric emptying and leading to a more sustained release of nutrients compared to whey protein.^60^ The specific amino acid composition and surrounding food matrix, such as dairy matrix components, are relevant features contributing to amino acid digestibility and postprandial whole-body protein metabolism.^61^ Overall, the degree of protein denaturation and amino acid composition dictate the rate of proteolysis and availability of amino acids in the small intestine.^62^

### Food matrix effects, digestion and absorption kinetics, and potential implications for the human gut microbiome

In the following subsections, we discuss how the food matrix shapes physiological response to food along the length of the gastrointestinal tract, providing evidence of food matrix effects on bioavailability and accessibility of nutrients. Then, we address the potential implications of these findings for microbial substrate availability and fermentation kinetics along the length of the gastrointestinal tract.

#### The oral phase

In the oral cavity, mastication serves to reduce the particle size of food, which is the initial step for digestion. Chewing mechanically breaks food into small fragments, providing greater surface area for enzyme access.^63^ Different food matrices are mechanically digested to different particle sizes before swallowing, and there is inter-individual variability in chewing mechanics, which produces differences in bolus particle sizes.^64^^,^^65^ These mechanical-level variations have direct implications for glycemic response as they impact initial accessibility of starch to digestive enzymes. Notably, differences in chewing also lead to differences in rate of microbial fermentation.^64^^,^^66^

In humans, chemical digestion of carbohydrates is initiated by *α*-amylase in the oral cavity. Higher levels of salivary *α*-amylase activity are associated with improved glycemic response to a carbohydrate load.^67^ While humans have a high level of salivary *α*-amylase activity, lingual lipase content is low, limiting the extent of oral chemical lipid digestion. The inverse is true in murine models, which have high lingual lipase but low *α*-amylase activity.^68^ Therefore, human studies focused on the oral phase of the food matrix effect are predominately focused on carbohydrates.

Simple carbohydrates (mono- and disaccharides) are rapidly fermented by the oral microbiota, reducing the pH at the site of fermentation.^69^ Depending on oral hygiene and individual microbiome composition, the acidic conditions within dental plaque can contribute to the development of dental caries. This process is understood to be mediated by cariogenic pathogens such as *Lactobacillus fermentum*, *Veillonella,* and *Prevotella,* among others.^70^^,^^71^ While the link between simple carbohydrates and caries is well established, how much of an impact, if any, dietary patterns have on microbial composition of the oral cavity has not been fully elucidated.^72^

#### Potential implications of the food matrix on the oral microbiome

We hypothesize that the food matrix dictates substrate availability for the oral microbiota by modulating the release of simple carbohydrates. Supporting this, in vitro evidence demonstrates that individuals with higher salivary *α*-amylase activity exhibit greater hydrolysis of cooked starch compared with those with lower enzyme activity.^73^ This results in higher concentrations of low–molecular–weight carbohydrates (disaccharides to octasaccharides), which are readily available for use by the oral microbiota.^74^^,^^75^ No difference in hydrolysis was observed for raw starch. This finding suggests that the dense crystalline matrix of native starch granules confers some resistance to enzymatic degradation in the oral cavity.^73^ Investigating how food matrices and carbohydrate complexities influence oral cavity microbial fermentation is predicted to be an important area of future diet‒microbiome research.

#### The gastric phase

Digestion in the gastric phase is largely characterized by enzymatic breakdown and denaturation of exogenous proteins. Digestive kinetics at this stage are impacted by thermal processing, type of protein, particle size, and presence of lipids. For instance, milk, an aqueous emulsion composed of protein, lipids, carbohydrates, and minerals, is vulnerable to modification when treated with high temperatures. Pasteurization and high-temperature treatment (72 and 140 °C, respectively) produce softer, more porous protein coagula. Increased porosity then allows for greater pepsin access, leading to more rapid protein hydrolysis.^60^ Further, the specific amino acid profile of proteins impacts gastric emptying due to differences in solubility, which delays digestion rates as measured by leucine absorption.^76^

In addition to enzymatic digestion during the gastric phase, peristaltic movement of the stomach plays a critical role in lipid absorption through mechanical emulsification. This process also mixes gastric secretions with digest, allowing gastric lipase and pepsin greater access to triglycerides and polypeptides for digestion, respectively.^77^ Mechanical and enzymatic action preprocesses the food matrix, maximizing the surface area available for lipase-mediated hydrolysis by reducing lipids into progressively smaller droplets. Larger particle sizes, higher viscosity, and a more intact food matrix delay emulsification and gastric emptying, modulating nutrient delivery to the small intestine.^78–81^

#### Potential implications of the food matrix on the gastric microbiome

The human gastric microbiota is a distinct microbial ecosystem compared with the oral cavity and esophagus and varies widely across individuals.^82^^,^^83^ A primary driver of this variability is the presence or absence of *Helicobacter pylori*. In *H. pylori*-positive subjects, the gastric mucosa is dominated by this species, reducing the relative abundance and diversity of the overall community.^83^^,^^84^ To date, studies aiming to characterize microbial communities in the gastric phase have focused on differences between healthy subjects and distinct patient groups including gastric carcinoma and gastritis.^82^^,^^85^ Evidence from cross-sectional studies suggests that dietary patterns rich in simple carbohydrates and sweets are associated with H. *pylori* colonization.^86-88^ Conversely, studies suggest intake of solid fats and cheese may be associated with a decreased risk of colonization.^88^ Regardless, there is a current dearth of literature evaluating how 1) the food matrix impacts the composition and function of the microbiota in the upper gastrointestinal tract and 2) gastric microbiota may impact subsequent digestion and absorption kinetics.

### Lower gastrointestinal tract

The small intestine is the principal site for major digestion and absorption. Here, mucosal and pancreatic enzymes, in addition to bile salts and other pancreatic secretions, facilitate the hydrolysis and solubilization of accessible nutrients for absorption into the intestinal mucosa. Physiologically, the colon plays an important role in the absorption of water and electrolytes.^77^ In addition, it harbors the greatest microbial density and represents the primary site of microbial fermentation of nutrients that escape digestion in the small intestine. Therefore, understanding what structural factors promote resistance to digestion, particularly before the colon, has important implications for substrate availability for microbial fermentation.

#### Particle size

Food can be conceptualized as a matrix of particles. Reduction of particle size through milling (e.g. grains), mechanical tissue disruption (e.g. ground meat), or the progressive emulsification of lipids increases the surface-area-to-volume ratio, facilitating enzymatic digestion and absorption.^19-21^ Digestion kinetics vary according to particle size and are often measured as biochemical responses postprandially. Consumption of wheat porridge containing large particles of wheat (2 mm) reduced the postprandial incremental area under the curve (iAUC) of blood glucose, insulin, C-peptide, and glucose-dependent insulinotropic polypeptide (GIP) compared to the small particle wheat porridge (<0.2 mm particles).^89^ Large particle chickpea flour (1.4–1.8 mm) also reduced glycemic response compared to finely milled chickpea flour.^90^ In a study that did not observe differences in digestion kinetics when comparing coarse and finely milled flour, the results are likely explained by the presence of a small proportion of fine particles within the coarse flour (up to 20% of particles smaller than 0.18 mm), which may provide sufficient rapidly digestible starch to attenuate the effects of larger particle sizes.^91^ Therefore, particle size can influence host postprandial physiological responses, even when driven by a relatively small fraction of particles. This highlights the importance of considering particle size distribution, rather than average particle size alone, when interpreting dietary effects.

The effect of particle sizes extends to whole foods like nuts as well. Large particles of almonds and peanuts, the result of either reduced mastication or minimal processing, seems to increase the incretin release, satiety, and fecal fat excretion, while reducing hunger sensation.^3,92–94^ Moreover, the consumption of amino acids versus whole proteins also results in more rapid absorption.^95^ For example, minced beef is more rapidly absorbed than intact steak because of mechanical disruption of muscle fibers.^96^ Mechanical reduction of particle size in navy bean flour was also found to increase protein digestibility in gastric and intestinal in vitro models.^97^

Altogether, these results suggest that large particle size, regardless of the dominant macronutrient, are more likely to escape absorption in the small intestine and reach the colon. Once these particles reach the colon, microbial fermentation is expected to depend, at least in part, on the relative surface area of each particle. However, as the fermentative metabolism occurs intracellularly, the relationship is unlikely to follow a direct single-step mechanism and may be affected by additional factors. For example, metabolite production is particle size dependent.^98^ The authors also found that divergent microbial structures favored a specific particle size: *Ruminococcaceae* and *Porphyromonadaceae* favored small particles*, Bacteroidaceae* favored medium particles, and *Coprococcus* and *Coriobacteraceae* favored large particles. These findings demonstrate how quantifying available nutrients provides an incomplete picture of diet‒microbiome interactions by overlooking the physiochemical state of microbial substrates from dietary sources.

#### Cell wall integrity or encapsulation

The plant cell wall, or other artificial encapsulation, acts as a physical barrier between nutrients and digestive enzymes. When left intact, intercellular structures are highly resilient to host digestion. Natural variations in cell wall structure, like porosity, have demonstrated the ability to either promote or restrict the diffusion of *α*-amylase and lipases, modulating postprandial metabolic responses.^99^^,^^100^ Mechanical manipulation (such as stirring or grinding), however, can physically break the cell wall, simulating similar impacts on digestion and absorption.

The physiological impact of cell wall integrity was demonstrated in a study comparing chickpea cotyledon cells processed to different extents: intact, isolated, or broken.^101^ In vitro, meals with broken cells were rapidly hydrolyzed by pancreatic *α*-amylase, whereas intact cells remained largely resistant to enzymatic action.^101^ When applied in vivo, meals containing broken chickpea cells elicited greater peaks in postprandial glucose and insulin compared to intact cells.^101^ Further, when cells remained intact the timing and degree of hormonal responses were shifted with a more sustained release of satiety hormones like GLP-1 and PYY reflective of a delayed nutrient release and extended satiety period.^101^ Mechanistic evidence from in vitro models using either intact or homogenized chickpea cells resulted in different rates of starch digestion (>20% in intact cells and 40% in homogenized cells), supporting these findings.^34^

For lipids, the cellular encapsulation or stabilized biopolymer networks similarly restricted absorption in the small intestine. Use of whole almond seeds or larger almond particles compared to almond oil as muffin ingredients resulted in lower postprandial triglyceride measures in humans.^102^^,^^103^ This finding suggests that the lipids remained trapped within the almond’s cellular matrix, limiting intestinal absorption. Taken together, these results demonstrate that cell wall integrity and encapsulation limit macronutrient bioaccessibility, delaying or reducing absorption in the small intestine.

#### Processing-induced structural changes

Beyond particle size reduction and cell wall disruption, food processing can induce modifications to the internal organization of the food matrix. These changes alter the physicochemical properties of macronutrients and their molecular interactions. For example, while prolonged thermal processing generally facilitates greater *α*-amylase diffusion across the cell wall resulting in increased starch accessibility,^104^^,^^105^ other processes can alter structures in a way that promotes resistance to enzymatic hydrolysis.^106-108^

The impact of modifications to the internal organization of the food matrix are particularly evident in bread production. Variations in kneading time, for example, can impact the glycemic index of bread; extending kneading from 10 to 15 minutes strengthens the gluten network, resulting in a firmer structure and a lower blood glucose response.^91^ Similarly, bioprocessing techniques like fermentation and germination can reorganize the matrix, leading to lower postprandial glucose and insulin responses compared to refined white bread.^106-108^

For proteins, processing can increase bioaccessibility by removing interfering structures. Dehusking chickpeas, for instance, increases soluble protein content while decreasing fiber, thus increasing protein bioavalibilty.^109^ Together, these processing-induced changes may enhance or reduce the access to intracellular nutrients, and ultimately influence digestion kinetics.

These matrix-driven changes in accessibility may also influence microbial activity in the small intestine and colon. The small intestinal microbiota appears to preferentially utilize simple carbohydrates, although there is considerable interindividual variability in the capacity to ferment more complex substrates.^110^ Evidence from ileostomy studies further supports the presence of a metabolically active and diet-responsive ileal microbiota.^111^^,^^112^

Thermal processing, through compounds such as dAGEs and LOPs, may also affect the composition of residing microbial communities.^47^ An in vitro digestion and fermentation study using pooled human fecal samples showed that heat treatment of starchy foods (banana and bread) impacted the microbiome, while heat-treated non-starchy foods (chicken and pepper) were not associated with major changes to the microbiota.^113^ In addition, boiled chickpeas produced microbial profiles more similar to starchy foods, while grilled chickpeas were closer to non-starch foods.^113^ These results may be explained by structural changes during high heat treatment, where starch molecules participate in Maillard reaction, and form dAGEs, thereby altering substrate availability for microbial utilization. In animal studies, consumption of a diet rich in dAGEs and LOPs reduced the gut microbial diversity and richness.^114^^,^^115^ Considering that 10% to 30% of consumed dAGEs are absorbed, these results suggest that both dAGEs and LOPs may act as fiber-like products, escaping digestion and absorption to reach the distal part of the gastrointestinal tract and be used as a substrate for fermentation by the microbiome.^116^

#### Resistant fractions

The formation of resistant fractions, such as dietary fibers, resistant starch, and resistant protein, also alters the digestion kinetics of macronutrients. For example, consumption of resistant starch results in a lower glycemic response postprandially after consuming reheated pasta^117^ and parboiled rice.^118^ Similarly, the consumption of proteins with resistant starch or fiber matrices reduces host absorption rates and increases the delivery of dietary protein to the colon, as indicated by higher nitrogen excretion.^119-123^

Small differences in the structure of dietary fibers can produce distinct microbial responses. A randomized clinical trial comparing structurally distinct sources of type 4 resistant starch (RS4), including crystalline maize, cross-linked tapioca, and cross-linked potato, demonstrated that even within the same class of resistant starch, the response of the microbiome is dose- and source-specific.^124^ Notably, *Eubacterium rectale* increased in all fecal samples for all subjects (*n* = 10) after RS4 in the form of crystalline maize was consumed, but no response was noted to cross-linked tapioca. On the other hand, in vitro, RS4 in the form of tapioca starch was effectively utilized by *Bifidobacterium adolescentis* and *Parabacteroides distasonis,* but not by *E. rectale*. This suggests that specific ester linkages in the tapioca matrix likely limit surface-attachment mechanisms of *E. rectale*, physically limiting its access to the substrate.^124^

Additionally, some researchers have introduced the concept of “resistant protein”, which further illustrates the matrix effect. Conceptually, this is similar to resistant starch, where the protein is entrapped inside intact plant tissue and resists intestinal proteolysis.^125^ In contrast, animal-derived protein matrices are more porous and lack similar fibrous barriers, requiring less kinetic energy for microbial acquisition of amino acids. However, modeling protein as a microbial substrate is inherently complex due to the presence of endogenous protein (e.g. mucin, digestive enzymes) that accompany unabsorbed dietary residues into the colon.^126^^,^^127^ Utilization of these endogenous protein sources is highly context dependent; for example, a deficiency of fermentable substrates can increase mucin breakdown.^8^

## Discussion

**Figure 2.**
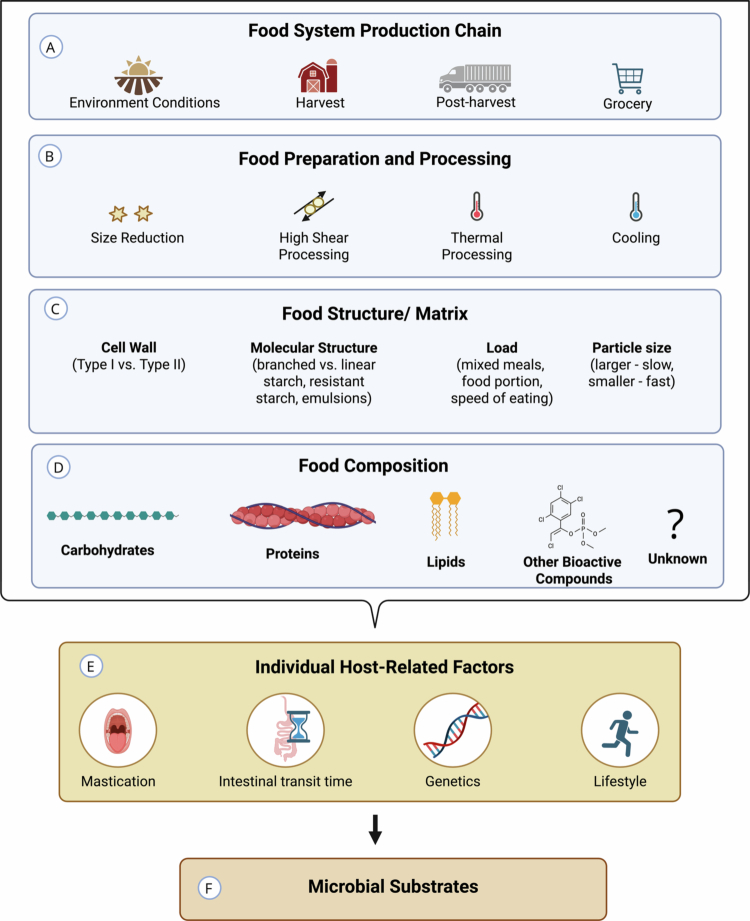
Food variable considerations in diet–microbiome research. A) The food system production chain, while not a primary focus of this paper, includes environmental and logistical variables (e.g. soil quality, harvest timing, storage) that impact nutrient quantity, stability, and accessibility of foods. B) Food preparation and processing involves mechanical and thermal methods, such as milling, high shear processing, cooking, cooling, and re-heat, fermentation, etc. These methods impact the food matrix with implications for host digestion kinetics and microbial substrate. C) Food matrix properties, including cell wall integrity and molecular structure, as well as variables like meal load and particle size, impact nutrient bioavailability and digestion in the gastrointestinal tract. D) Food composition details nutrients and bioactive compounds within the matrix whose accessibility is influenced by (E) individual host-related factors, which include factors like intestinal transit time, genetics, stress, all of which ultimately dictate (F) microbial substrates.

This narrative review aimed to achieve three primary objectives: 1) summarize how food processing impacts digestion kinetics, 2) illustrate the role of the food matrix in determining substrate availability, and 3) identify current knowledge gaps in diet‒microbiome research. The reviewed literature consistently demonstrates that food matrix features such as particle size, cell wall integrity, and molecular arrangement are important modulators of nutrient digestion and absorption. These structural variables, therefore, also dictate the amount and physical form of substrate made available to the gut microbiota. Figure 2A–F provides a high-level overview of food variables that influence substrate availability to the gut microbiota. Further, while the literature was more limited, findings also reflect food matrix effects on microbial fermentation kinetics.

Gut microbiota research is often focused on the microbes present in stool, which represent the microbiome within the lumen of the distal colon, but, while notably less dense, a microbiome is present along the entire gastrointestinal tract. Microbial fermentation may occur in more proximal segments, such as the stomach or small intestine, thereby shaping the microbial substrate availability in the colon.^110^^,^^128^ Pathological conditions such as small intestinal bacterial overgrowth (SIBO) provide an example of how microbial activity in the small intestine can alter the composition of the luminal bolus and modulate nutrient availability for both host and colonic microbiota.^129^ However, direct investigation of the small intestinal microbiota remains methodologically challenging. Current assessments frequently rely on ileostomy effluent samples, which are difficult to obtain and present translational limitations.^110^ Individuals with ileostomies exhibit altered intestinal physiology and microbial ecology compared to individuals with an intact gastrointestinal tract. Further, experimental conditions, such as purification of the effluent from other substrates prior to the analysis, may not fully represent the native *in vivo* microbiota.

In addition, while this review used macronutrients to illustrate the fundamental role of food structure on digestion and absorption kinetics, it is essential to recognize that macronutrients are simply one component of a complex food matrix. Other bioactive compounds (e.g. vitamins, minerals, phytonutrients) have their own distinct kinetics and are likely to influence gut microbiome composition and function. For instance, the gut microbiota metabolizes plant-derived flavonoids into bioactive compounds and utilizes minerals like iron as enzymatic cofactors.^130^^,^^131^ Presence of additives such as emulsifiers (e.g. lecithin, carboxymethylcellulose, and monoglycerides) can further complicate diet‒microbiome interactions by altering the food matrix and subsequent digestion kinetics.^132^^,^^133^ Nonetheless, the structural principles discussed (i.e. effect of the food matrix on macronutrient absorption) provide a foundational framework that needs to be considered when endeavoring to advance the understanding of diet‒microbiome interactions.

### Knowledge gaps and challenges in current research

Table 1 provides an overview of the current knowledge gaps and challenges as identified by this review. The challenges were 1) individual differences in digestive physiology, 2) developing a multidisciplinary food matrix framework, 3) capturing the spatiotemporal dynamics of the food matrix in vivo, and lastly, 4) sampling shortcomings limiting total gut microbiome documentation. For all challenges, related sources were identified to provide background context and similar research examples. In addition, research questions that acknowledge the current gaps are provided to inspire future research. The following narration concludes these knowledge gaps.

**Table 1.** Current challenges and potential future directions: investigating the food matrix effect in vivo.

Challenges	Background	Research questions	Similar research and study design ideas
Challenge 1: individual differences in digestive physiology	Nutrition research requires a movement towards personalization.^134^^,^^135^ It is also accepted that microbial features typically reflects the physiological state of the host.^136^ Following, there are a number of documented interindividual differences with ingestion and digestion of food, including: mastication,^64^ gastric emptying,^137^ transit time,^134^ digestive enzyme activity, satiation, osmotic pressure and pH of gastrointestinal tract, and hormonal excretions. Further, temporal symptoms such as anxiety and stress alter intestinal transit, often facilitating faster transit times.^138^^,^^139^ To what degree these individual responses impact ecological conditions for residual microorganisms is unknown.	- To what extent do inter- and intraindividual responses to food ingestion influence microbial diversity? - To what extent do different microbial communities influence inter- and intraindividual responses to food ingestion? - Do microbes favor specific host genetics?	*Make interindividual variability the endpoint in RCT*^134^*; measuring defined physiological limits of the host. | Similar concept: Constrained-Disorder Principal*^135^*; measuring the dynamic adaptability of individuals' microbiomes. | Gut physiology and environment are key variables to understand gut microbial composition and digestion*^136^*; intra-individual variations are reflected in stool moisture and fecal pH, while inter-individual variations are reflected in transit times and pH.*
Challenge 2: developing a multidisciplinary food matrix framework	Food intake is predominantly quantified through nutrient analysis software that computes food intake at a nutrient level. However, this logic presupposes nutrients are being consumed from the same food matrix. Accounting for food matrix components, such as particle size, cell composition, viscosity, and solubilization, are relevant to all diet research, but particularly when studying diet‒microbiome relationships. Therefore, there is a need to develop a food matrix framework that allows for the inclusion of food matrix features in diet‒microbiome models.	- Can food be deconstructed into different component levels, such as particles and cellular fragments? - How can food matrix components be computationally mapped? - Does food contain an average predictable matrix composition? What are those factors?	*Similar work: food performance and matrix-aware guidance in Farm-to-Fork agenda*^140^*; which also advocated for longer RCT lengths to inform the creation of 'matrix-based dietary guidelines'. | Similar ideas: Kiousi and others advocate for interdisciplinary collaboration in food matrix and gut microbiome research, particularly regarding the food science field.*^141^ *| Identifying intact cellular components of food vs fragmented cellular parts (quantifying ratio between them; alpha and beta diversity of cellular food components). Deconstruct and identify food's cellular and fragmented parts > use information to computationally reconstruct food.*
Challenge 3: capturing the spatiotemporal dynamics of food matrix digestion in vivo	Food matrices can be engineered to leverage specific in vivo effects in functional food products, such as decreasing absorption barriers to enhance intestinal absorption of bioactive nutrients.^142^ This could also be leveraged to modify microbial access and fermentation throughout the gastrointestinal tract, especially targeting microbial fermentation in the colon. Estimating spatiotemporal dynamic of the food matrix is primarily limited to in vitro studies. Nasogastric tubes and catheters introduced transnasally have been used to analyze gastric aspirates and small intestinal samples, respectively, but these are highly invasive protocols.^143^ Other less invasive in vivo advances include NMR and magnetic resonance techniques.^144^	- How does food shape the ecological conditions of the gastrointestinal tract? - What technology can elucidate the food matrix effect in vivo? - How does the food matrix influence microbial colonization mechanisms?	*Similar work: CODY (COputing DYnamics of gut microbiota) by Geng and others, 2021.*^145^ *| MRI monitoring tracks food digestion at the particle level*^146^*; discusses in vivo ideas such as fast imaging, motion compensation, and high-performance magnetic gradient systems to investigate solid food digestion in the stomach. Also highlights difficulty in vivo studies present as they require fast motion-robust imaging systems with reduced voxel size that visually capture the volume of food ingested. | Gastric time technologies (old: ultrasonography,*^147^ *.new: wireless motility capsule.*^148^*)*
Challenge 4: sampling shortcomings limiting total gut microbiome documentation	The gastrointestinal microbiome has primarily been studied in the lumen of the distal colon (fecal samples) and the oral cavity (saliva or plaque samples). This is due to inherent challenges in sampling throughout the gastrointestinal tract. A recent advancement that may help overcome sampling difficulties are engineered capsules that collect intestinal fluid at specific intestinal segments.^149^^,^^150^	- What is the interaction of various microbial ecosystems along the length of the intestine? - What other sampling methods or technologies remain unexplored?	*Studies on H. Pylori suggest a crosstalk between gastric and* *intestinal microbiota, as well as altered gastrointestinal* *physiology that may have a complementary effect.*^151^

A major gap in diet‒microbiome research is the inability to accurately model kinetics of microbial substrate availability along the entire gastrointestinal tract (Table 1). Currently, researchers endeavoring to estimate microbial substrate availability need to infer from studies on host digestion and absorption kinetics, which overlook potentially metabolically active communities in the more proximal gastrointestinal tract.^152-154^ A heavy reliance on fecal sampling, which is highly practical and feasible, only provides a limited snapshot of the lumen of the distal colon, ignoring potential interindividual variability in fermentation in the small intestine and proximal colon.^155-157^

As new methodologies to improve understanding of the role of diet in changing the microbiome continue to be developed, the impact of food structure remains a critical confounding variable (Table 1). For example, there is increasing interest in the use of food DNA markers in stool to assess dietary intake.^158^ However, given the effects of food structure on digestion and absorption kinetics, and that fermentation is more rapid with small particles and varies between individuals, the structure of food consumed, along with physiological variables such as chewing, gastric pH, transit time, among others, will dictate how much recognizable food DNA markers are present in stool. To advance the understanding of diet‒microbiome interactions, a multipronged methodological approach is necessary (Table 1). Dietary assessments should be combined with laboratory food analysis to determine the initial structure of the diet and fecal food DNA markers to measure what was unused. Further, advanced in vitro and ex vivo assays, including models like the simulator of the human intestinal microbial ecosystem (SHIME) and gut-on-a-chip and gut-in-a-dish systems, can be used to isolate the specific impacts of the food matrix on the gut microbiome.^159^ Ultimately, combining these diverse methodologies will be critical in the development of strategies to more accurately model microbial substrate availability. Regardless of the approach, developing approaches to account for food matrix effects is critical to advance the field of diet‒microbiome research.

### Strengths and limitations

This narrative review integrates a broad range of evidence from multidisciplinary fields and outlines key challenges and future directions for assessing the effects of the food matrix *in vivo*. Additional strength is the application of the SANRA approach to support the quality and transparency of the review. However, the use of a nonstructured search strategy may introduce selection bias. To minimize this, studies were selected as comprehensively as possible according to predefined inclusion criteria and agreed upon by the authors. Future research should also summarize the impact that other dietary components have on gut microbiota outside of macronutrients.

## Conclusion

The complexity of the food matrix and its impact on digestion, absorption, and microbial fermentation kinetics requires a paradigm shift in how we approach dietary intake data for diet–microbiome research. The evidence outlined here demonstrates that food structure is a crucial determinant of both host nutrient bioavailability and microbial substrate availability. To move the field forward, the development of novel methodologies that account for food structure is recommended. Integrating food matrix variables will improve our ability to model and predict the complex relationship between the human diet and the gut microbiome.

## Supplementary Material

Supplementary Material_Food Matrix.docx Supplementary Material_Food Matrix.docx
